# Production of precision slots in copper foil using micro EDM

**DOI:** 10.1038/s41598-022-08957-9

**Published:** 2022-03-23

**Authors:** Katerina Mouralova, Josef Bednar, Libor Benes, Tomas Plichta, Tomas Prokes, Jiri Fries

**Affiliations:** 1 Faculty of Mechanical Engineering, Brno University of Technology, Brno, Czech Republic; 2 Faculty of Mechanical Engineering, Jan Evangelista Purkyně University, Ústí nad Labem, Czech Republic; 3 Department of Production Machines and Design, Technical University of Ostrava, Ostrava, Czech Republic; 4 Institute of Scientific Instruments of the CAS, V. V. I., Brno, Czech Republic

**Keywords:** Electrical and electronic engineering, Engineering

## Abstract

Electrical discharge machining (EDM) is an unconventional machining technology. It allows machining of at least at least electrically conductive materials. The trend of miniaturization of industrial products is obvious. However, the required quality and accuracy must be maintained, which can be achieved with micro-EDM. One of the industrial products is also optical devices used for testing cars. These contain miniaturized parts, which are, however, necessary for their proper functioning. For this reason, this study was performed, which focused on the production of a precise slot measuring 5000 × 170 µm in a copper foil with a thickness of 125 µm. The same copper foil was used as a tool, which represents an advance in the production of micro-parts.The use of the same semi-product for the production of the slit as well as the tool itself has not yet been presented in any similar study. A design of experiment Box and Behnken Response Surface Design was performed for a total of 15 rounds, monitoring the effect of machine setting parameters (Pulse current, Pulse on time and Voltage) on responses in the form of Erosion rate, corner radius, slot length and width. Using multi-criteria optimization, the optimal setting of the machine parameters for the production of a given slit was determined, which is Pulse current = 2.1 A, Pulse on time = 40 µs and Voltage = 238.8 V. Micro-EDM technology has been found to be suitable for the production of miniaturized slits.

## Introduction

Micromachining is generally characterized as the production of parts with dimensions less than 1 mm^1^. Due to the growing demands for product miniaturization in many areas of industry, it is necessary to constantly increase the demands on individual production technologies so that these parts can be produced with the required precision and quality. Technologies fully adapted for the production of micro-parts include unconventional technologies of electrical discharge die-sinking machining (EDM)^2,3^. EDM makes it possible to machine all at least minimally electrically conductive materials, regardless of their mechanical or physical properties. The principle is the thermoelectric removal of the workpiece material into the shape of the used tool electrode in the presence of the working medium—dielectric liquid (most often kerosene). In this way, it is possible to create even thin-walled profiles of very soft materials, because no classical mechanical forces act on the workpiece, as is the case with conventional machining. The disadvantage of this technology is its relatively high energy intensity, which can be significantly reduced by optimizing a particular production process^4^.

Thanks to the increased energy intensity of EDM technology, it is necessary to carefully optimize the production process of each part so that not only the maximum possible saving of machine time but also so that the manufactured part has the required accuracy and quality. For this reason, an extensive study was performed containing the design of experiment, which optimized the production of the micro-part of the slit from the copper foil using the same copper foil as the tool electrode. The novelty of this study lies mainly in the use of the same material for the workpiece and for the tool, all in micro dimensions. This new solution thus brings comfort to the manufacturers of these miniaturized parts, who do not have to buy a semi-finished product for the product and tool separately. They only buy copper foil, which they use both for the product itself and as a machining tool. This study builds on previous studies on EDM, such as the corner wear electrode study^5^ or the study on defects occurrence while machining steels 1.2363 and 1.2343 ESR^6^. The results of this study can be used not only in the production of a key part for optical devices designed for testing car lights, but also for the production of other parts from thin films.

This study focused on the production of a slit measuring 5000 × 170 µm (tolerance at the length of the slit is ± 60 µm and at a width of ± 5 µm) from copper foil with a thickness of 125 µm. In practice, slits are used in optical instruments to reduce the total amount of light that results in a narrow collimated beam (parallel light rays), which is used in further analysis. Due to the high infrared radiation (heat loss power) of the light sources used today (xenon lamp with a power input of several kW) in optical devices, it is necessary that the slit material has good thermal conductivity and mechanical resistance. In our case, the copper foil intended for the workpiece will also be used as a tool—an electrode. The width of the slit depends on the type of light source used and the device for which it is intended. These optical instruments are mainly used for testing car headlights.

### Literature review

Lim^7^ studied the machining of high-aspect ratio micro-structures which were divided into two processes using the micro-electro-discharge machining. The two processes contained the on-machine fabrication of the micro-electrodes with a high-aspect ratio, and the second—the EDM of the workpiece in the micrometre range. During the experiment by employing different methods in order to investigate and make a thin electrode of the desired measures, for this purpose an optical sensor was developed. Zhang^8^ focused on the precision machining of micro tool electrodes during the micro EDM process while drilling array micro holes. In order to improve the on-line machining accuracy of micro electrode two methods were combined, i.e. a tangential feed WEDG method and the on-line measurement employing a charge coupled device, which helped to obtain a precise diameter of micro-electrode. For improving the machining efficiency of micro electrodes the combination of the TF-WEDG method and a self-drilled holes method was used. Ay^9^ investigated the best factors and level conditions to optimize the micro-electrical discharge machining drilling process of the nickel-based superalloy Inconel 718. For the experiment, the gray relational analysis method was used and the machining parameters, such as pulse duration and discharge current were taken into account. The results showed that the pulse current was more efficient on performance characteristics than the pulse duration. D’urso^10^ studied the process performance of micro-EDM drilling of stainless steel plates. Varied several process parameters were used during the experiment, such as voltage, peak current and frequency, using the electrodes made of two different materials: tungsten carbide and brass. During the drilling process, the material removal rate and tool wear ratio were studied. Plaza^11^ performed an experimental study focusing on the micro EDM drilling of Ti6Al4V by the helical electrode. The parameters studied in the experiment were electrode wear, material removal rate, micro-hole quality and machining time. They designed a new strategy due to an inefficient removal of debris while increasing hole depth. Somashekhar^12^ studied the numerical simulation of the micro EDM model with multi-spark, which was based on the finite volume method and was developed to solve the micro-EDM model equations, and consequently, predict the effect of spark ratio, i.e. spark on and off time, on the temperature distribution in the material. The results obtained were successfully tested against published ones. Shao^13^ focused on the comprehensive electro-thermal modelling of the crater formation in micro-EDM in order to simulate the process of crater formation. That model included realistic machining conditions such as Gaussian distributed heat flux, expending plasma and temperature dependent thermal properties. The simulation results obtained showed a good agreement with experimental results. Liu^14^ studied the effects of the surface layer of AISI 304 on micro EDM performance, mainly the effect of three surface layer of austenitic stainless steel. For the experiments, machining multilayer stainless steel workpieces with and without surface treatment were employed. The results revealed that the steel workpiece with oxidation treatment has the highest surface free energy and the highest material removal rate in comparison with those without treatment and with acid pickling treatment. Hourmand^15^ tried to develop new fabrication and measurement techniques of micro-electrodes with a high aspect ratio for micro EDM, which were based on horizontal moving block electrical discharge grinding and gage block using a typical EDM machine. Yu^16^ developed a micro punching system with a micro electrical discharge machining module, and the micro punch was produced using reversed electrode. The micro die and the reversed electrode were prepared using micro EDM milling. The results showed that after punching there was no damage on the edges of the micro die and micro punch. In order to optimize process parameters, many techniques are used, including genetic algorithms, such as in the publications by Matousek^17,18^.

## Experimental setup and material

**Figure 1:**
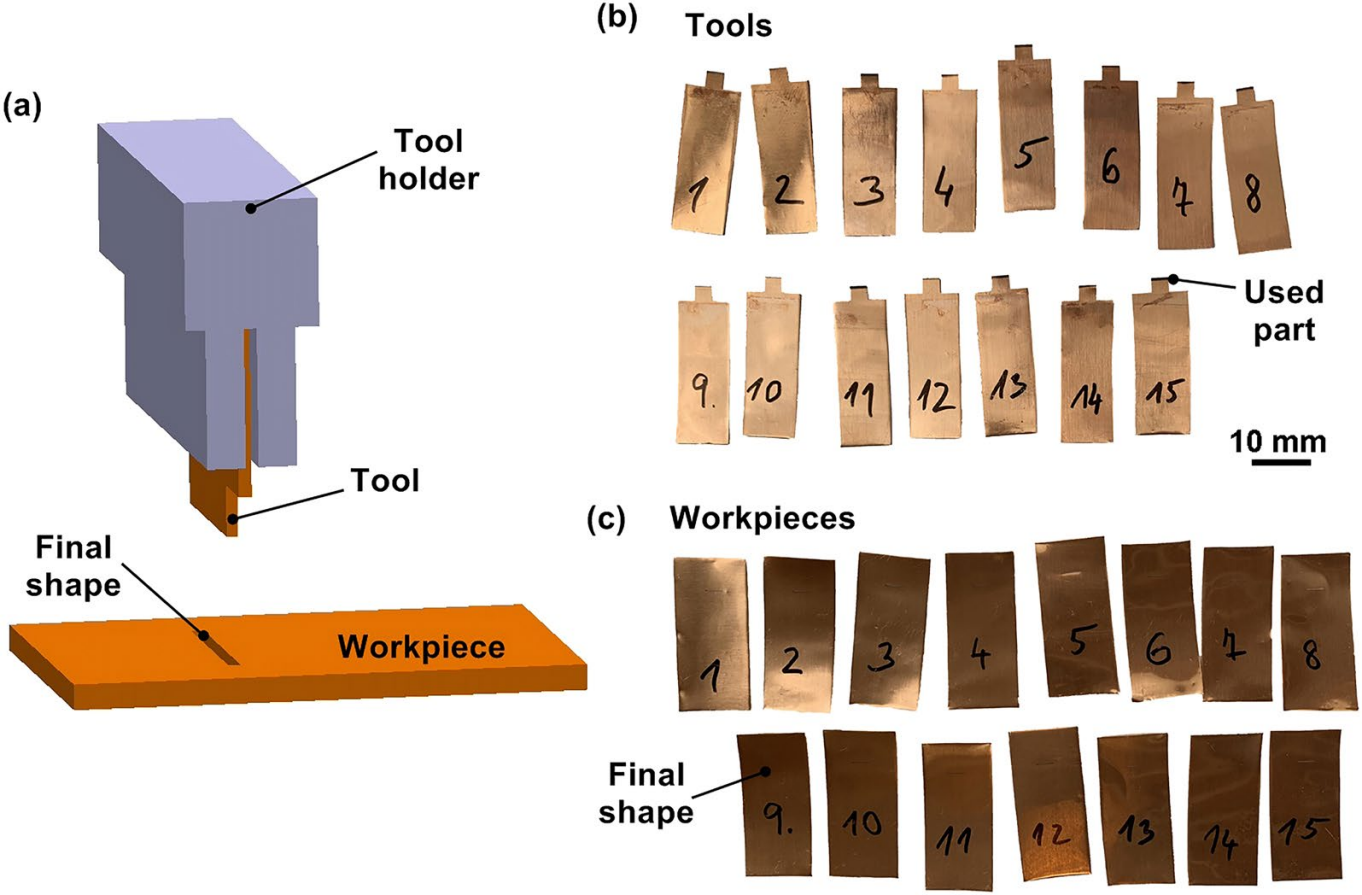
Description of the performed experiment with individual tools and workpieces (**a**) schematic representation of the experiment (created in program SolodWorks-www.solidworks.com), (**b**) tool electrodes used, (**c**) workpieces with eroded final slot shape.

The samples for the experiment shown in Fig. 1c were made of 0.125 mm thick copper foil, the electrodes used are shown in Fig. 1b. These electrodes were cut to the required size (always to an eroding edge length of 48 mm) clamped in a specially made holder shown in Fig. 1a using an α-C600iB type wire electrical discharge machine (WEDM) from FANUC. WEDM cutting of the electrode proved to be necessary because the corners of the electrode were tested using conventional shears. Furthermore, the tested corner was not able to erode the slit in the required shape and always caused the curvature of the eroded slit.

All samples were made on a 433GS electrical discharge die-sinking machine supplied by PENTA and equipped with a P-MG1 generator. During machining, all samples were immersed in kerosene.

The design of experiment performed in this study was based on monitoring the influence of three independent machine setting parameters, which were: voltage (*U*), pulse current (*I*), pulse on time (*T*_*on*_). The pulse off time (*T*_*off*_) parameter was fixed at 20 µs in all rounds of the experiment. The machining input parameters are listed in Table 1, and their limit values have been determined based on extensive previous tests. It was assumed that some response surfaces would be curved, so the design of experiment was designed as a Box and Behnken Response Surface Design. It is designed to model a full quadratic regression model, where the quadrates of individual input parameters describe the curvature. In addition to the quadrates of the input parameters, the regression model contains linear terms describing trends and second-order interactions describing the interaction of two parameters. If the examined area of the input parameters after standardization is viewed as a 3-dimensional cube, the responses will be measured in the centres of the edges of this cube. Furthermore, it was measured 3 times at the central point in order to capture the variability of the measurement. The relationship between Box and Behnken Design and commonly used Central Compossite Design is described in detail statistically in Montgomery^19^, with Box and Behnken Design being more efficient because coefficients of the same regression model can be obtained with fewer measurements. The data collection plan itself is described in Table 1, the order of measurements is not standardized, but it is randomized to avoid systematic bias of the experiment due to external influences. The investigated responses will be erosion rate and geometric accuracy parameters of shapes.

**Table 1:** Machining parameters used in the experiment and cutting speed.

Number of sample	Pulse current (A)	Pulse on time (µs)	Voltage (V)
1	2.6	70	280
2	1.9	70	220
3	1.2	40	220
4	1.9	70	220
5	1.9	100	160
6	2.6	70	160
7	1.2	70	160
8	2.6	100	220
9	1.9	40	280
10	1.2	70	280
11	1.2	100	220
12	1.9	100	280
13	1.9	70	220
14	2.6	40	220
15	1.9	40	160

## Results and discussion

### Experimental methods

The studied samples were studied using a Lyra3 type electron microscope (SEM) from TESCAN. Using this microscope, all assessed dimensions of the slit, i.e. its length, width and also the radii in the corners, were gradually measured. An approximation circle was used to measure the radii.

### Statistical evaluation

**Figure 2:**
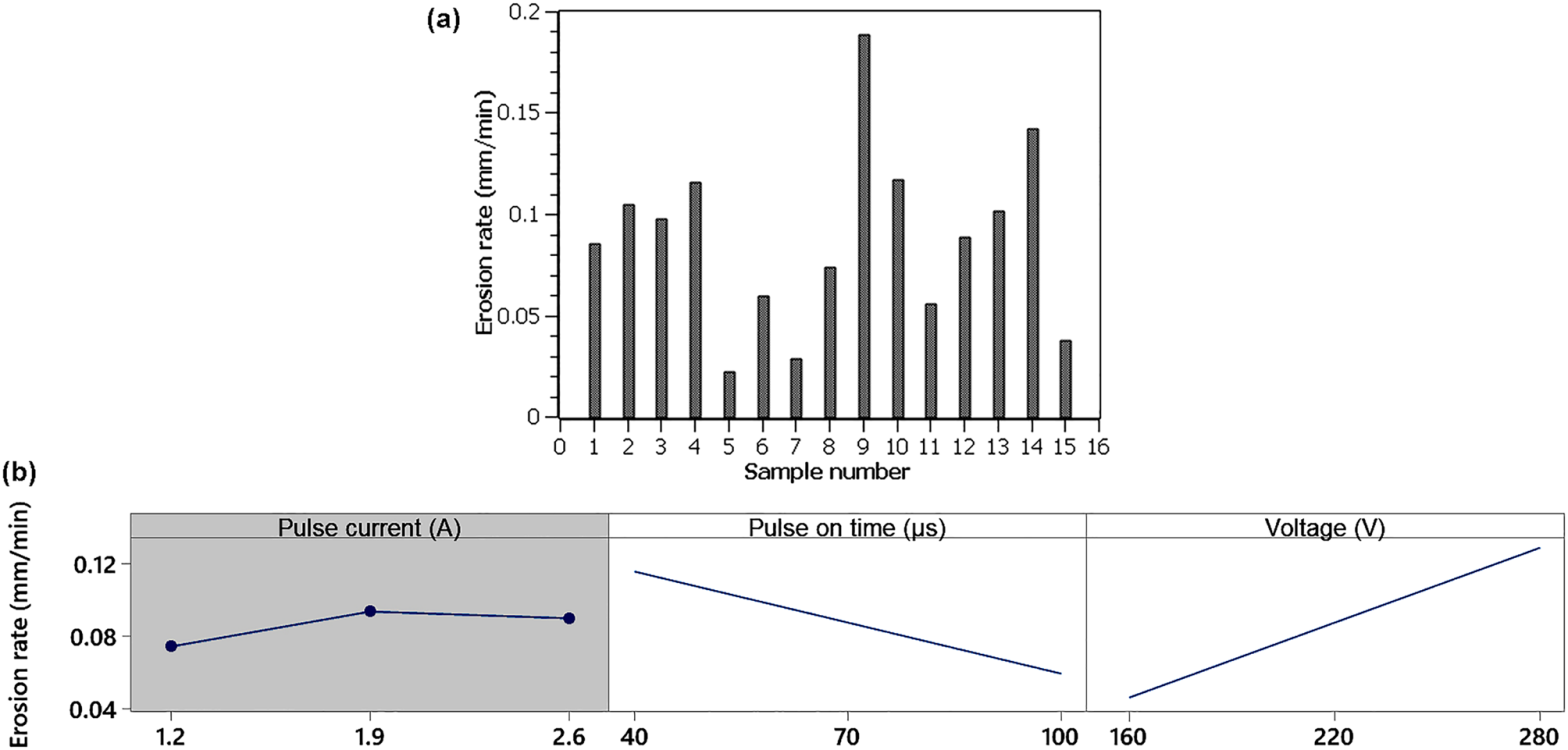
(**a**) Eroding rate of individual samples, (**b**) main effect plots for erosion rate (A gray background represents a term not in the model, which is not statistically significant and is therefore not included in the model).

The eroding speed of the EDM process is determined by the machine parameters and cannot be easily set in the program as in conventional machining. The EDM machine must constantly maintain the gap between the tool and the workpiece, otherwise a short circuit would occur. For this reason, the eroding rate was described from the machine display throughout the whole design of experiment, and for each sample, this rate was plotted in Fig. 2a. It can be seen from this graph that the eroding rate differed relatively significantly with the change in machine parameter settings. The fastest eroded speed was for Sample 9 with the setting of machine parameters *I* = 1.9 A, *T*_*on*_ = 40 µs, *U* = 280 V at a speed of 0.19 mm/min. On the contrary, the slowest eroding rate was recorded for Sample 5 and only of 0.02 mm/min. The highest erosion rate achieved here (0.19 mm/min) is relatively high compared to other micro-EDM studies. These are mainly Singh^20,21^ or Kurnia^22^ studies, but unfortunately the same material was not machined or a wall electrode was used as in this study.

Based on the measured data, fully quadratic regression models were compiled, from which insignificant members (significance level of 0.05) were removed by the Stepwise method while maintaining the hierarchy of the model. The hierarchy of the model preserves insignificant factors in the model if their square is significant. In addition to the regression equations themselves, which will be used for multicriteria optimization, the coefficient of determination R^2^ was always determined, which indicates what percentage of the variability of the observed response is described by the regression model.

Erosion rate is affected by only two factors Pulse on time and Voltage. Pulse on time has a negative effect and Voltage has a positive effect, as can be seen in Fig. 2b. The regression equation describing the dependence is:

1 $$Erosion\; rate = 0.0018 - 0.00094\;T_{on} + 0.000689\;U.$$

Although only 2 factors were significant, regression Eq. (1) describes 72.69% variability in the observed data (R^2^ = 72.69%).

**Figure 3:**
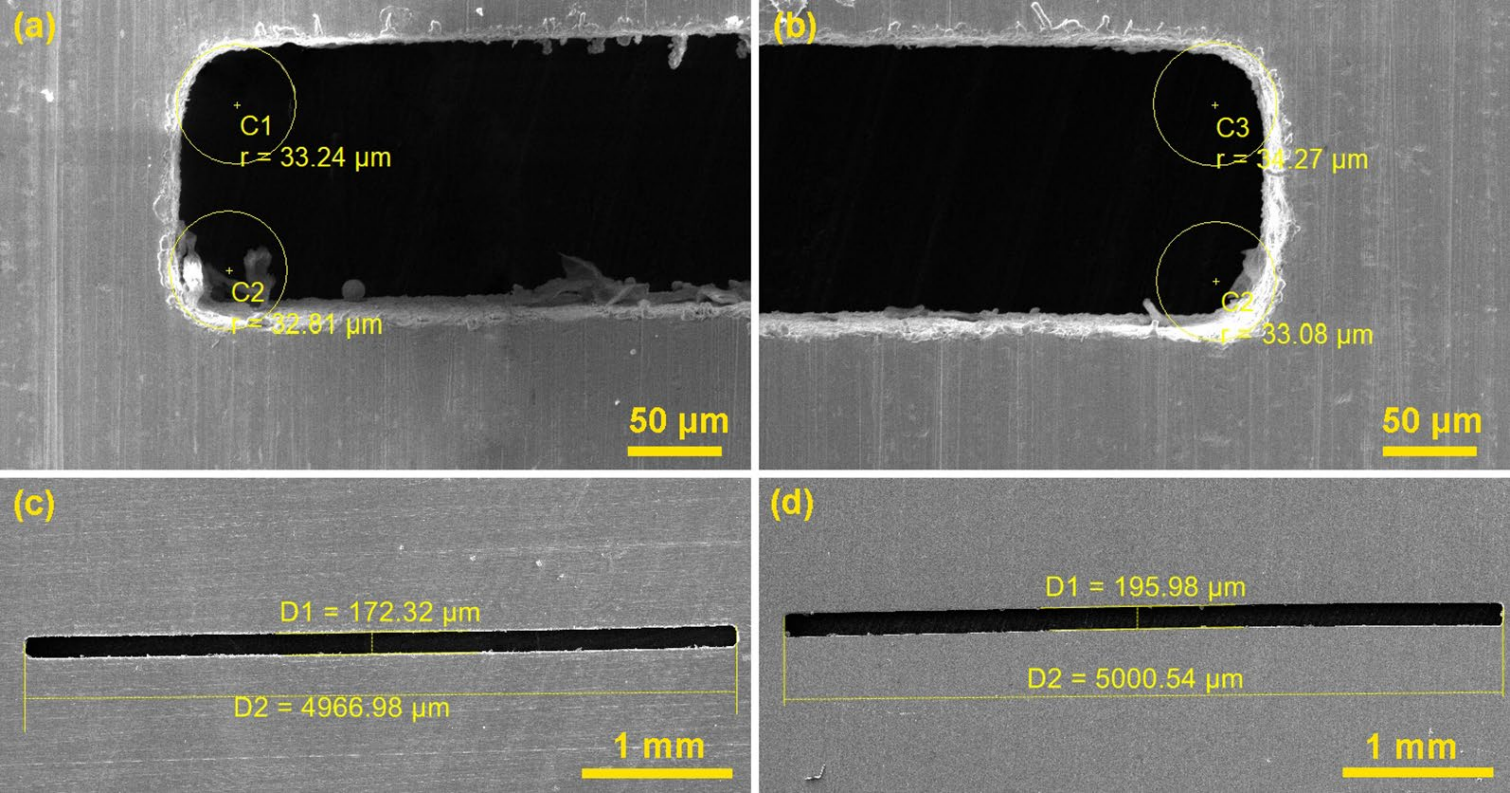
(**a**, **b**) Corners of Sample 4 (edited in program Adobe Photoshop www.adobe.com/cz/products/photoshop.html), (**c**) Sample 2 with the most accurate width, (**d**) Sample 6 with the most accurate length.

Due to the fact that the most accurate dimension of the slot is 5000 × 170 µm and also the minimum values of the radii in the corners were accurately measured using an electron microscope and the results processed into Table 2. The smallest radii were achieved for Sample 4, the most accurate slit width for Sample 2 was 172.32 µm and the most accurate length for Sample 6 was 5000.54 µm. These best samples are shown in Fig. 3. It is therefore clear that for no sample the most accurate parameters were achieved at the same time, therefore a statistical evaluation of the performed design of experiment in terms of the accuracy of the produced shapes is necessary.

**Table 2:** Dimensions of individual slit shapes.

Number of sample	Edge 1 (µm)	Edge 2 (µm)	Edge 3 (µm)	Edge 4 (µm)	Length (µm)	Width (µm)
1	55.24	54.95	56.19	56.56	4927.97	155.41
2	42.57	39.76	39.6	39.75	4966.98	172.32
3	40.33	40.56	39.41	40.4	4973.74	199.36
4	33.24	32.81	34.27	33.08	4994.01	159.03
5	40.76	40.12	41.56	38.92	4977.63	166.93
6	40.63	41.03	45.05	43.11	5000.54	195.98
7	35.5	36.28	38.75	38.92	5006.05	165.81
8	51.07	49.59	55.35	55.67	5000.77	203.64
9	40.15	42.44	39.22	43.52	4784.52	179.28
10	43.69	44.91	44.43	42.81	4989.97	189.22
11	42.86	41.06	40.09	42.63	4953.05	181.14
12	42.07	44.21	43.52	44.26	4922.87	178.96
13	36.13	34.94	37.28	36.13	4948.75	167.93
14	44.82	44.17	48.44	46.74	4997.15	158.72
15	35.26	35.76	37.08	34.22	4990.92	198.4

**Figure 4:**
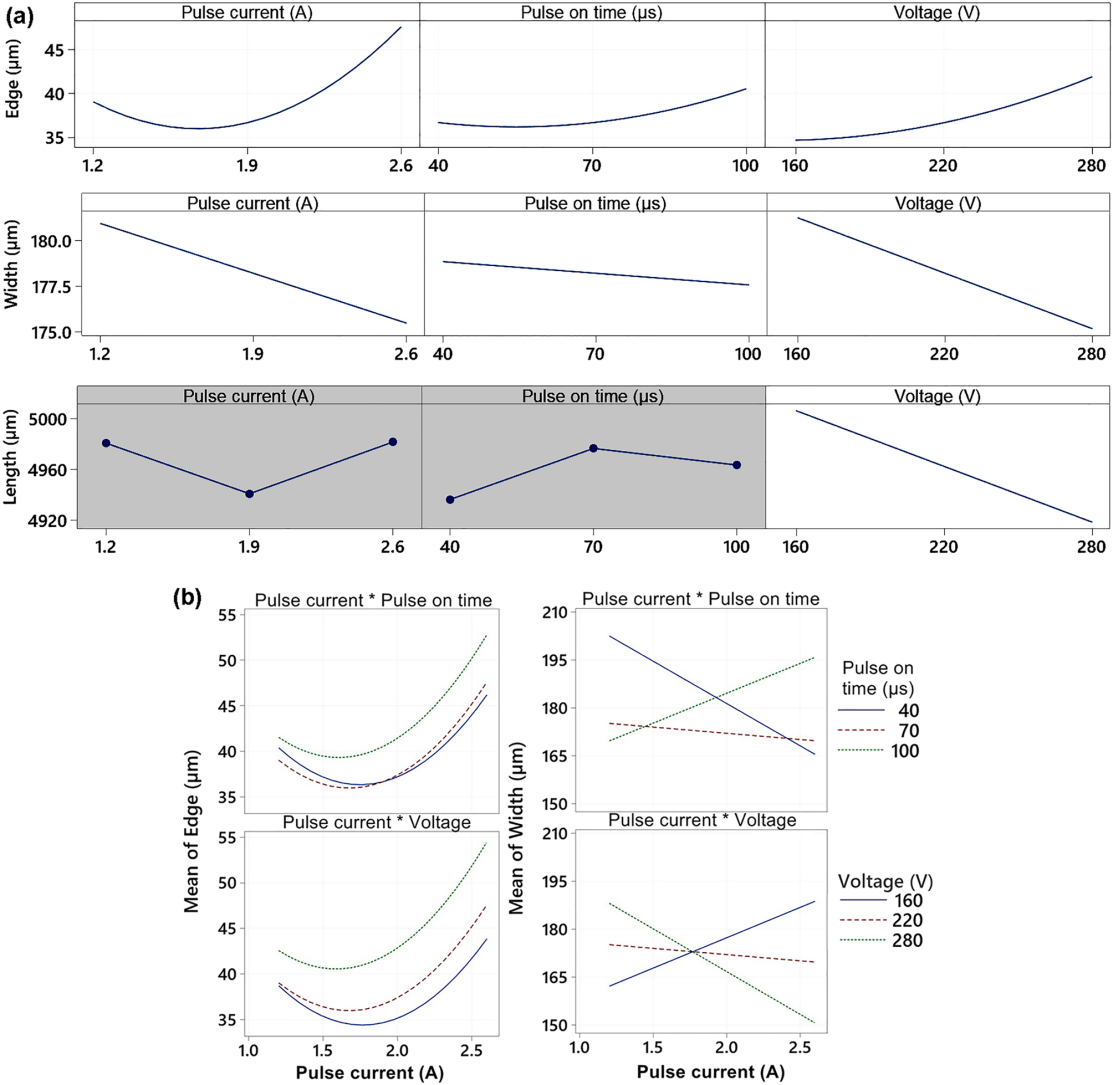
(**a**) Main Effect Plots for Edge, Width and Length (A grey background represents a term not in the model, which is not statistically significant and is therefore not included in the model) (**b**) Interaction plots for significant interactions.

In contrast to the previous response, which was the Erosion rate in the radius dimension equation, all squares and factor interactions, with the exception of the Pulse on time × Voltage interaction, were significant. The action of the input factors themselves and their squares is shown in Fig. 4a, while the action of the interactions is shown in Fig. 4b. The regression equation itself is:

2 $$\begin{aligned} Edge & = 113.7 - 58.53I - 0.359T_{on} - 0.2130U + 13.52I^{2} + 0.002152T_{on}^{2} + 0.000449U^{2} + \hfill \\ & \quad + 0.0642I \cdot T_{on} + 0.0398I \cdot U. \hfill \\ \end{aligned}$$

**Figure 5:**
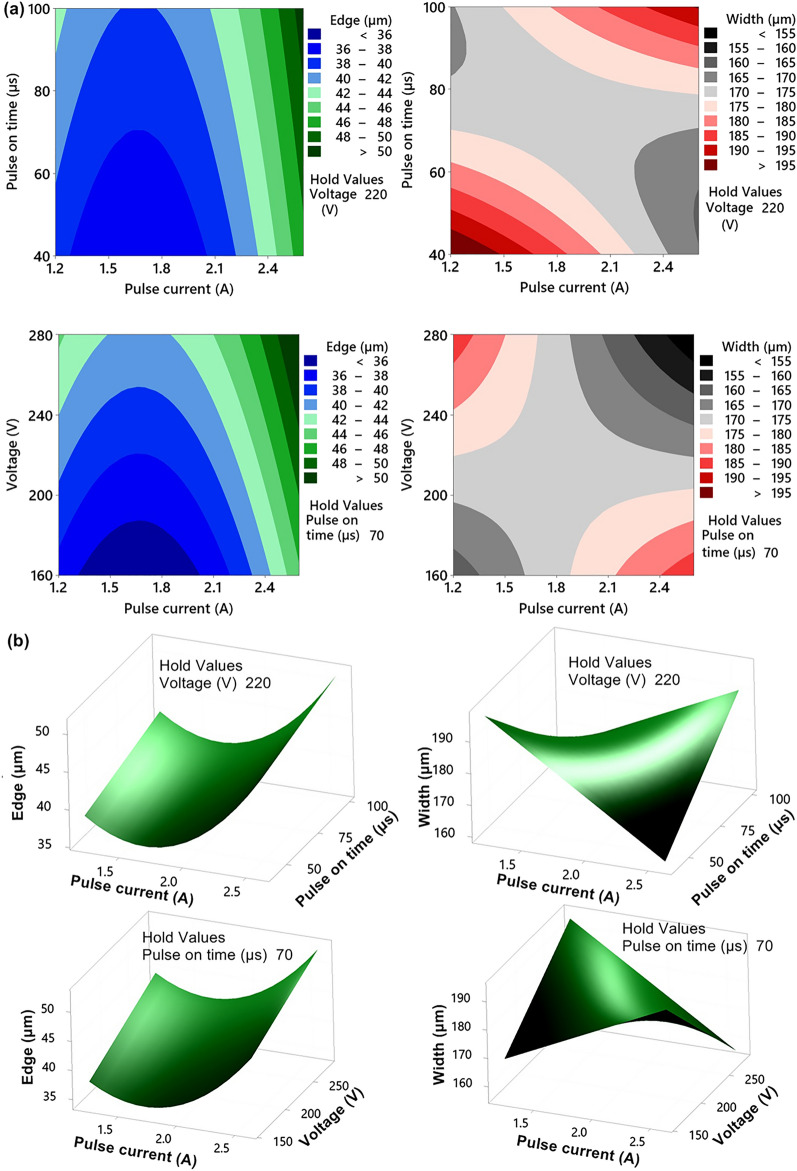
Response areas of responses, where there is a significant interaction and the coordinate not displayed is fixed at the central level (created in program MiniTab-www.minitab.com) (**a**) 2D, (**b**) 3D.

This Eq. (2) was shown in Fig. 5, as blue-green 3D Contour plots, so that the last variable not shown was fixed at the central level. The coefficient of determination R^2^ = 87.34%, so the model describes about 87% of the variability of the observed response.

The width of the slit is mainly affected by the interactions Pulse current × Pulse on time and Pulse current × Voltage, which are shown in Fig. 4b and their effect can be seen nicely in Fig. 5, where 3D sections of the Width response surfaces are displayed in grey-red. The squares of all three factors are insignificant. Regression Eqs. (3):

3 $$Width = 139 + 27.3\;I - 1.449\;T_{on} + 0.673\;U + 0.752\;I \cdot T_{on} - 0.381\;I \cdot U,$$

describes about 60% of the variability of the monitored response (R^2^ = 59.58%), while the required slit width is 170 µm.

The length of the slit is affected only by the Voltage factor (p-value = 0.02 < 0.05), as nationalized in Fig. 4a. The other members of the regression model were insignificant. The model itself describes about 35% of the variability of the monitored data (R^2^ = 35.07%), and it can be assumed that the length is also influenced by other factors that have not been monitored, and now their effect manifests itself as a residual error of the model, while the equation is:

4 $$Length = 5122.8 - 0.729U.$$

### Multicriteria optimization

**Figure 6:**
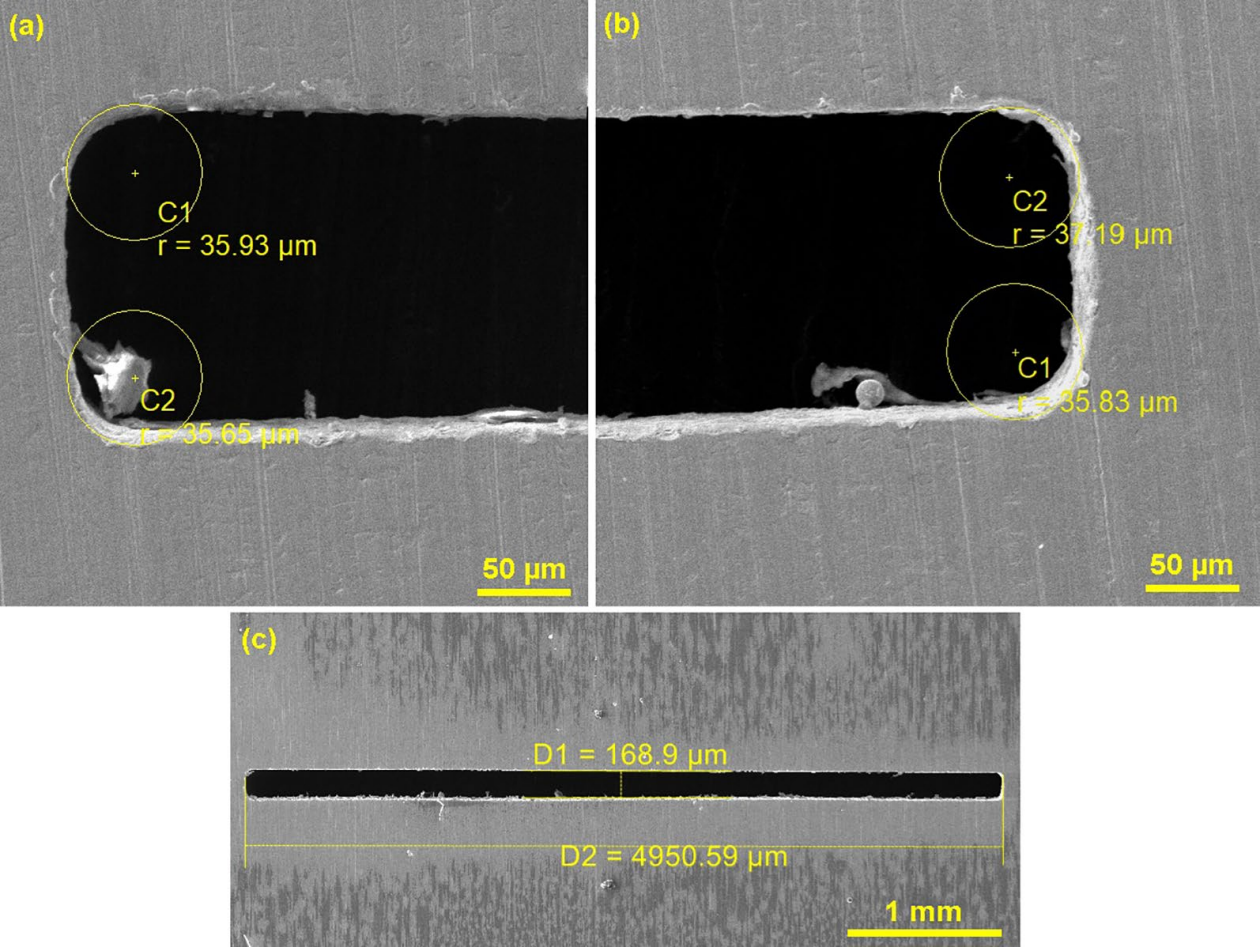
Slot made on the basis of optimized parameters from multi-criteria optimization (edited in program Adobe Photoshop www.adobe.com/cz/products/photoshop.html) (**a**, **b**) slit corners, (**c**) whole slit.

As usual, there is a technical contradiction in EDM machining, which has been studied in several other publications, such as EDM machining of steels 1.2363 and 1.2343 ESR^6^ or WEDM machining of Mg/CRT/BN composites^23^, titanium alloy 6242^24^ or titanium (α–β) alloy^25^. Because if the Erosion rate is maximized, the Edge will be minimized, the Width will be targeted at 170 µm and the Length will be targeted at 5000 µm, the input parameter setting requirements will be conflicting, so multi-criteria optimization should be used. Multi-criteria optimization was used to find the optimal process settings, which is integrated directly into the Minitab statistical software. Linear desirability functions were chosen in the whole range of monitored responses. Because the main requirement is mainly for a slot width of 170 µm and a minimum Edge in the corners, these characteristics were given Importance 2. The eroding rate is less important than the above shape characteristics, so Importance was left at the default level 1. The length depends only on the Voltage parameter, by maximizing which we achieve an elongation of the slit, but the radius in the corners will increase and the slit will be widened, therefore the Importance of length 0.5 was chosen. In addition, the length can be changed by making a different length of the machining electrode. The output of the procedure was the optimal setting of input parameters and estimation of the value of individual responses (Fit), which are given in Table 3. These input parameters were rounded to the resolution level of the machine and on the basis of this setting a real product was produced, the individual responses (Real) of which are given in Table 3. The slit thus produced is shown in Fig. 6, while the eroding rate decreased slightly, but smaller radii at the corners were achieved. The only parameter that differed negatively from the estimate in real terms (Fit) was the width, which decreased to 168.9 µm.

**Table 3:** Multiple response prediction.

Variable	Setting	
Pulse current (A)	2.1	
Pulse on time (µs)	40	
Voltage (V)	238.8	

Response	Fit	Real
Width (µm)	170.06	168.9
Erosion rate (mm/min)	0.128	0.113
Length (µm)	4948.8	4950.59
Edge (µm)	40.01	36.15

## Conclusions

In order to optimize the production of a slit in a 125 µm thick copper foil, which should have dimensions of 5000 × 170 µm, the design of experiment Box and Behnken Response Surface Design was performed. The eroding tool used was the same foil, which is also the material being machined. During the experiment, the influence of the machine setting parameters, which were Pulse current, Pulse on time and Voltage, on the responses in the form of Erosion rate, radius in the corners, length and width of the slit was monitored. Based on the performed measurements and statistical evaluations, the following conclusions were reached:

- The fastest eroded sample was Sample 9 with the setting of machine parameters *I* = 1.9 A, *T*_*on*_ = 40 µs, *U* = 280 V at a speed of 0.19 mm/min, on the contrary, the slowest erosion rate was recorded in Sample 5 with the setting of machine parameters *I* = 1.9 A, *T*_*on*_ = 100 µs, *U* = 160 V and only of 0.02 mm/min.
- Erosion rate is affected by only two factors Pulse on time and Voltage, where Pulse on time has a negative effect and Voltage has a positive effect.
- The smallest radii were achieved in Sample 4, the most accurate slit width in Sample 2, namely 172.32 µm and the most accurate length in Sample 6, namely 5000.54 µm, from which it is clear that no sample achieved the most accurate parameters at the same time.
- An equation describing the radius dimension was created, in the model all squares and factor interactions were obtained with the exception of the Puls on time × Voltage interaction significant.
- The width of the slit is mainly influenced by the interactions Pulse current × Pulse on time and Pulse current × Voltage.
- The length of the slit is only affected by the Voltage factor.
- Using multi-criteria optimization, the optimal setting of the machine parameters for the production of a given slit was found to be *I* = 2.1 A, *T*_*on*_ = 40 µs and *U* = 238.8 V, and based on this setting a slit was produced that met all accuracy requirements (the tolerance on the length of the slit is ± 60 µm and the width ± 5 µm).

Based on the above conclusions, it can be clearly stated that the performed multi-criteria optimization ensured the production of the slot in the given accuracy, using the same copper foil for the workpiece and for the tool itself. It will be possible to produce this slot only at the cost of the foil, it will not be necessary to order material separately for the manufactured part and separately for the tool needed for production.
